# Effect of Water Suppression and Metabolite Cycling on Quantification of ^1^H MRS Spectra in the Human Brain at 3 Tesla

**DOI:** 10.1002/mrm.70352

**Published:** 2026-03-19

**Authors:** Dinesh K. Deelchand, Pierre‐Gilles Henry

**Affiliations:** ^1^ Center for Magnetic Resonance Research and Department of Radiology University of Minnesota Minneapolis Minnesota USA

**Keywords:** brain, metabolite cycling, semi‐LASER, VAPOR

## Abstract

**Purpose:**

This study investigates the effect of VAPOR water suppression and metabolite cycling on metabolite quantification and macromolecules in proton magnetic resonance spectroscopy.

**Methods:**

Single‐voxel semi‐LASER spectra (*T*_R_/*T*_E_ = 3000/28 ms) and metabolite‐nulled spectra (macromolecules) were acquired in five healthy subjects in the posterior cingulate cortex at 3 T using two different water suppression schemes: VAPOR or metabolite cycling (MC). Post‐processed spectra were quantified using LCModel. Metabolites concentrations were compared between the two schemes.

**Results:**

Region specific differences in macromolecule resonances were observed and the concentration of most metabolites was significantly higher when using MC compared to VAPOR. The difference was most pronounced for total creatine (−14% with VAPOR vs. MC, *p* < 0.05).

**Conclusion:**

The macromolecules spectrum included in the LCModel basis set must be measured with the same water suppression scheme as the metabolite spectrum for accurate quantification.

## 1 Introduction

Proton magnetic resonance spectroscopy (^1^H MRS) enables noninvasive measurement of various metabolites in the brain. The concentration of the most concentrated neurochemicals, for example, *N*‐acetylaspartate (NAA), total creatine (tCr), total choline (tCho), glutamate (Glu) (< 12 mM), is several orders of magnitude smaller than the water concentration (∼40 M) in brain tissue. Therefore, water suppression (WS) is required to reliably measure and quantify these neurochemicals without baseline distortion or water sidebands from the intense water signal [1].

Several WS techniques have been proposed for localized ^1^H MRS sequence, including chemical shift selective (CHESS) [2], WS enhanced through *T*_1_ effects (WET) [3], variable power and optimized relaxations delays (VAPOR) [4] and most recently metabolite cycling (MC) [5]. Since its introduction in 1999, VAPOR has been widely used in both humans and animal brains. The VAPOR module consists of eight RF pulses interleaved with OVS pulses for human studies. It is the most commonly used WS technique due to being less sensitive to *B*_1_ inhomogeneities and *T*_1_ relaxation time variations than other water saturation schemes [1].

MC is also becoming a popular method to acquire metabolite spectra. It is a two‐shot technique where a single RF pulse with sharp transitions is used to invert the upfield and downfield regions without affecting the water signal. One benefit of MC is that shot‐to‐shot phase and frequency corrections are possible during post‐processing using the water signal even when the metabolite signal is too low on single shots (e.g., for small voxels) [6, 7].

Two recent MRSI studies compared VAPOR and MC WS techniques [8, 9]. One study reported significant differences in tCr/NAA between the two techniques, while *myo*‐inositol/NAA was slightly higher with MC (not statistically significant). Another study reported slightly higher concentrations for the major metabolites with MC, although these differences were also not statistically significant. Both studies either used simulated macromolecule (MM) or excluded MM contribution during data fitting which could affect accurate metabolite quantification. At present, it is unclear whether the use of MC versus VAPOR for WS influences macromolecule spectra. Therefore, the aim of the current study was to quantitatively compare the effects of VAPOR and MC on MM signals and metabolite concentrations in the human brain at 3 T using single‐voxel semi‐LASER MRS.

## 2 Methods

Five healthy subjects (47 ± 22 years old, 1 female) were scanned on a 3 T Siemens Prisma^fit^ scanner after giving informed consent. The study was approved by the institutional review board at the University of Minnesota.

The standard body coil was used for transmit and a 32‐channel head coil for signal reception. A volume‐of‐interest (VOI) of 20 × 20 × 20 mm^3^ was positioned in the posterior cingulate cortex (PCC) using *T*_1_‐weighted MPRAGE images (1 mm^3^ isotropic resolution; repetition time, *T*_R_ = 2530 ms; echo time, *T*_E_ = 3.65 ms; inversion time, *T*_I_ = 1100 ms; flip angle = 7°; GRAPPA acceleration factor = 2). First‐ and second‐order shims were adjusted in the VOI using FAST(EST)MAP [10]. Transmit power for localization and WS pulses was calibrated in each participant [11]. Localized ^1^H spectra were acquired using an optimized semi‐LASER sequence [11, 12] (*T*_R_/*T*_E_ = 3000/28 ms). Metabolites (64 averages) spectra were acquired with two different WS schemes: VAPOR or MC, with both modules interleaved with OVS pulses. Water reference scans were also acquired for eddy current correction [13] (RF pulses in VAPOR switched off) and for quantification (VAPOR and MC modules disabled). Metabolite‐nulled MM semi‐LASER data (*T*_R_/*T*_E_/*T*_I_ = 2500/28/750 ms) were also acquired with 128–256 averages in PCC in all subjects using VAPOR and MC techniques (Table S1). Due to the short *T*_1_ of MM at 3 T [14], the difference in *T*_1_‐weighting of MM resonances is negligible if *T*_R_ of 2.5 or 3 s is used to acquired MM spectra.

All MRS spectra were processed offline using the MRspa package [15] in MATLAB (The MathWorks Inc., USA). Shot‐to‐shot frequency and phase corrections were performed in addition to eddy current correction for both metabolite and metabolite‐nulled MM spectra (post‐processing steps described in Table S2). Note that six MM transients acquired using the MC technique were discarded due to corrupted water lineshapes. MM spectra from all participants were summed together for each scheme (VAPOR or MC). The tCr‐CH_2_ peak at 3.93 ppm was removed using a Hankel singular value decomposition algorithm in MATLAB.

Nineteen model basis set spectra were simulated based on density matrix formalism [16]: alanine, ascorbate (Asc), aspartate (Asp), creatine, γ‐aminobutyric acid (GABA), glucose (Glc), glutamate (Glu), glutamine (Gln), glutathione (GSH), glycerophosphorylcholine, *myo*‐inositol (Ins), *scyllo*‐inositol, lactate (Lac), NAA, N‐acetylaspartylglutamate, phosphocreatine, phosphorylcholine, phosphorylethanolamine (PE), and taurine (Tau). Two basis sets were generated; one containing the metabolite‐nulled VAPOR macromolecule (MM) spectrum and another one with the MC macromolecule spectrum.

Processed summed metabolite spectra were quantified with LCModel v6.3‐0G (Stephen Provencher Inc. Oakville, Ontario, Canada) using the built‐in water scaling option [17]. Metabolite spectra acquired using VAPOR were quantified using the VAPOR MM basis set while the MC datasets were quantified using the MC MM basis set. For comparison, the MC dataset was also fitted using the VAPOR MM basis set. No baseline correction, zero‐filling, or apodization functions were applied to the in vivo data prior to the analysis. Spectral fitting was performed from 0.5 to 4.2 ppm. Metabolite concentrations were corrected assuming a *T*_2_ of tissue water of 79 ms [18] while the effect of metabolite *T*_2_ relaxation was neglected. MPRAGE images were segmented using the FMRIB software library [19]. The CSF fraction in the VOI and a tissue water content of 82% were used to determine water‐referenced metabolite concentrations.

The reported signal‐to‐noise (SNR) was measured in MRspa as the peak height of NAA in the frequency domain divided by root mean square noise between −2 and −6 ppm, without any apodization applied to the data.

Only metabolites with a mean Cramer‐Rao lower bounds (CRLB) ≤ 20% for all WS schemes are reported. In addition, metabolites that were highly correlated to each other (*r* < −0.7) are reported as sums, for example, tCr (creatine + phosphocreatine), tCho (glycerophosphocholine + phosphocholine), and glucose + taurine. Metabolites' concentration between WS schemes were compared using paired student's *t*‐test. To account for multiple comparisons, *p* values were adjusted using the Benjamini–Hochberg false discovery rate method, and adjusted *p* < 0.05 was considered statistically significant. More details on MR data acquisition, processing and quantification are given in Table S2.

## 3 Results

The mean water linewidth measured from the PCC was 5.8 ± 0.6 Hz. High‐quality spectral data were acquired in all participants as reflected by the high SNR of the NAA: 232 ± 54 and 232 ± 56 for spectra acquired with VAPOR and MC, respectively.

**FIGURE 1:**
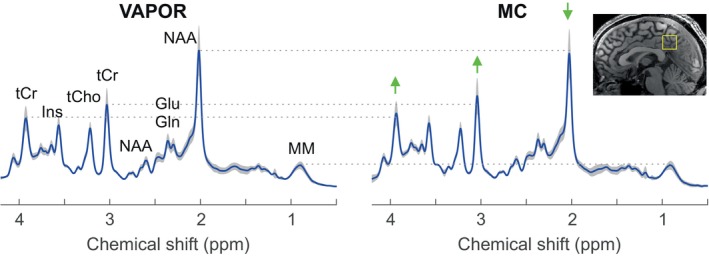
Mean (blue) and standard deviation (gray) of the semi‐LASER spectra (*T*_R_/*T*_E_ = 3000/28 ms) acquired from five subjects using two difference water suppression schemes: VAPOR (left) and MC (right). Using MC results in higher tCr singlet intensities compared to VAPOR, while NAA singlet is slightly reduced. For display purposes, a Gaussian weighting of 0.12 s was applied to all spectra. Insert shows the PCC VOI location on the anatomical image.

Figure 1 shows the mean semi‐LASER spectra acquired from all subjects, which clearly showed differences in the spectral pattern between the VAPOR and MC WS schemes. The tCr peaks were higher, most visibly at 3.03 ppm, in MC compared to VAPOR. In addition, the intensity of the NAA singlet at 2.01 ppm was lower when using MC (Figure S2A). No noticeable difference in the MM signals not obscured by metabolite signals between 0.9 and 1.9 ppm was observed between the two WS techniques. The fractions of white matter, gray matter and CSF in the PCC voxel were 14% ± 3%, 69% ± 7%, and 17% ± 8% respectively, across all subjects.

**FIGURE 2:**
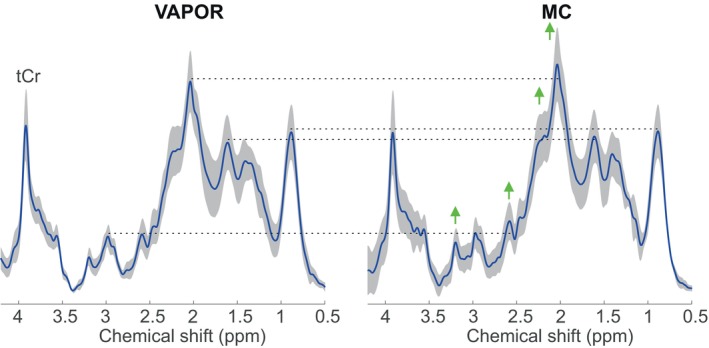
Mean (blue) and standard deviation (gray) of measured macromolecule signals acquired with semi‐LASER (*T*_R_/*T*_E_ = 2500/28 ms) from all five participants using VAPOR (1000 averages, left) and MC (994 averages, right) WS techniques. The summed MC and VAPOR spectra were scaled to match the 0.9 ppm peak, since no different in signal at this resonance was seen in Figure 1. Signals around 2 and 3.2 ppm exhibited higher intensities when using MC compared to VAPOR WS. Note that the tCr‐CH_3_ singlet at 3.93 ppm is present in both spectra due to longer *T*_1_ relaxation time of this resonance. It is typically removed to obtain the MM spectra included in the LCModel basis set. For display purposes, a Gaussian weighting of 0.12 s was applied.

Distinct differences were observed in the metabolite‐null macromolecule spectra measured with VAPOR and MC. Although the spectral pattern was comparable, most MM resonances were higher with MC than with VAPOR (Figures 2 and S2B), particularly in the regions between 2 and 2.6 ppm.

**FIGURE 3:**
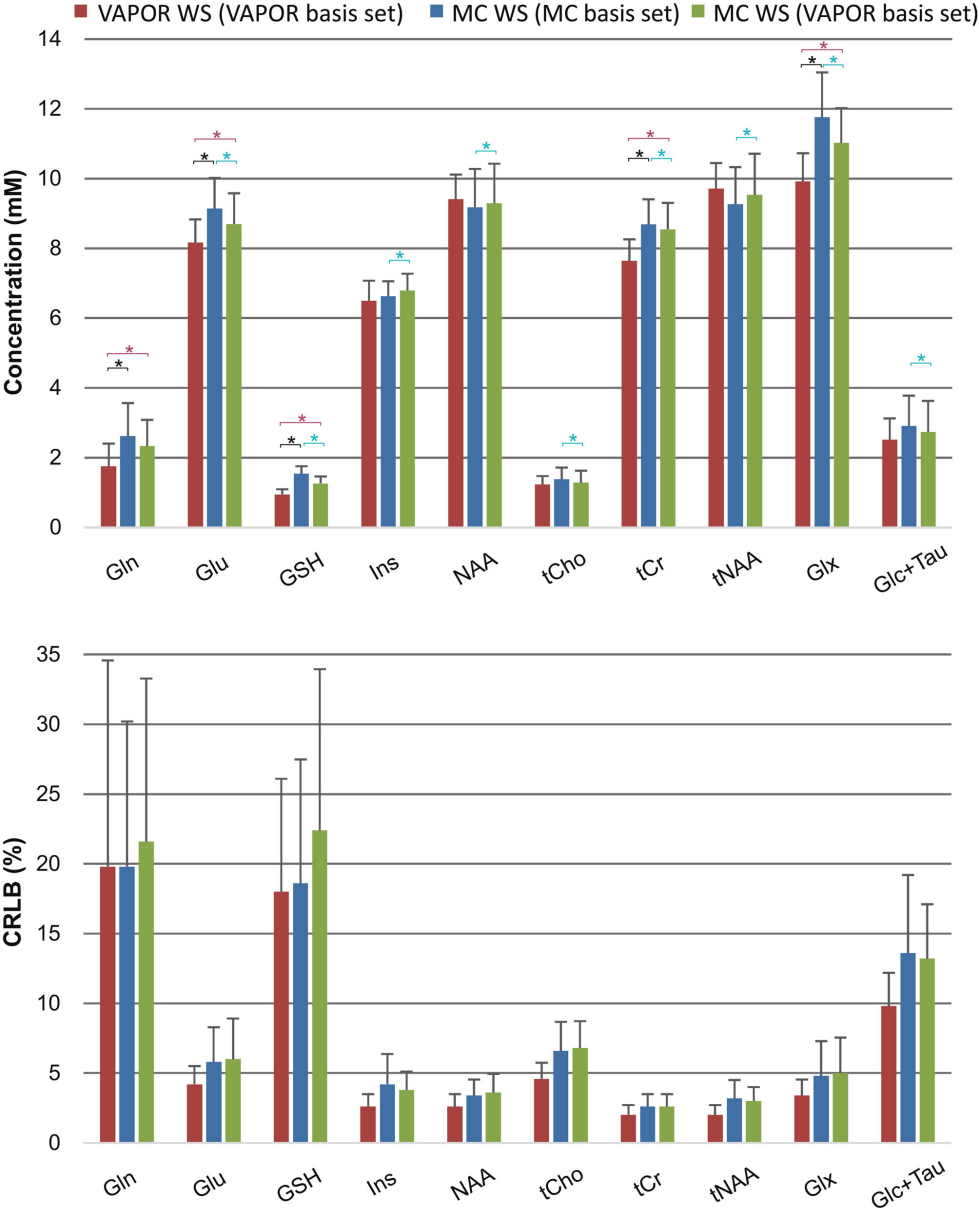
Mean concentration (mM) and CRLB (%) of metabolites measured from five participants. Metabolites data acquired with VAPOR WS and fitted using VAPOR MM were compared with metabolites data acquired with MC WS and fitted using either MC or VAPOR macromolecules. * indicates significance after FDR correction (adjusted *p* < 0.05) between methods. Error bars represent standard deviation.

Concentrations of neurochemicals with the different WS schemes and corresponding CRLBs are reported in Figure 3. When spectra were fitted with a basis set containing MM measured with the same WS scheme, comparison between MC and VAPOR showed higher concentrations (*p* < 0.05) with MC: glutamine (+49%), glutamate (+12%), glutathione (+62%), tCr (+14%), and glutamate+glutamine (Glx, +19%).

When MC metabolite spectra were fitted with a mismatched basis set (VAPOR MM basis set), concentrations of Glu, GSH, Ins, NAA, tCho, tCr, tNAA, Glx, and Glc + Tau were statistically lower (*p* < 0.05) than those obtained with the matched basis set (MC MM basis set) (Figure 3), showing that using a basis set with mismatched MM introduced bias in metabolite concentrations.

## 4 Discussion

The current study shows that five of the main metabolites (Gln, Glu, GSH, tCr, and Glx) had significantly higher concentrations when acquiring MRS spectra using MC compared to VAPOR for WS. In addition, the metabolite‐nulled MM spectra differed between the two WS techniques, but the differences were localized: MM signals were higher with MC than with VAPOR primarily in the 2.0–2.6 ppm region and near 3.2 ppm, while other resonances were similar. Fitting MC spectra with a mismatched basis set (VAPOR MM basis set) resulted in lower concentrations than fitting with a matched basis set (MC MM basis set), showing that MM spectra must be measured with the same WS scheme as the metabolite spectrum for reliable quantification.

The concentration of tCr was reduced by 14% when using VAPOR compared to MC WS. Several human and animal studies have also reported changes in the tCr signal when using off‐ and on‐resonance saturation pulses [8, 9, 20, 21, 22, 23]. This decrease in concentration was suggested to be related to the magnetization transfer (MT) effect through various mechanisms [20]. Contrary to the MC WS technique where the water signal is not affected, the VAPOR module consists of eight pre‐saturation pulses which affect the water magnetization. Multiple saturation of the water protons could lead to chemical exchange effects and cross‐relaxation, thereby reducing the tCr signal [21]. When a single WS pulse was combined with the MC WS technique to partially suppress the water signal, tCr concentration was comparable to using MC WS only (Figures S1–S3). This confirms that MT effects need a longer saturation time to become apparent.

Several *J*‐coupled metabolites, for example, Gln, Glu, and GSH showed lower apparent concentrations in the human brain when using VAPOR compared to MC WS. Similar changes in Glu and Gln levels due to off and on‐resonance MT effects were reported in the rat brain [21]. Glu, Gln, and GSH, like tCr, all have exchangeable protons such as hydroxyls, amides, and amines groups which are in exchange with the bulk water pool [24, 25] such that saturation of the water peak can affect their concentrations.

We believe this is the first study to report changes in macromolecule pattern with different WS techniques in the same brain region. Similar to the decrease in apparent metabolite concentrations observed with VAPOR, the localized decrease observed in the MM resonances with VAPOR is likely due to water‐mediated MT effects since tissue water protons exchange with mobile macromolecule protons [26]. A previous study reported that MT does not affect the MM signals in the brain [27] where an off‐resonance saturation pulse was used to produce the MT effect, contrary to direct water saturation used in the present study. In contrast, another study has reported potential saturation transfer effects between water and MM signals [28]. Further investigation is needed to determine the exact mechanism by which water affects the MM spectral pattern.

Changes in metabolites and MM were observed in the PCC voxel, which is primarily composed of gray matter in this study. Because metabolite [29, 30] and MM distributions [31, 32] differ between tissue types, the observed effects may not generalize to other brain regions. Future studies are needed to investigate regional variability and incorporate tissue segmentation to better account for gray and white matter differences.

## 5 Conclusion

The present study shows that using VAPOR WS results in lower apparent concentrations for several metabolites (notably Glu, Gln, GSH, and tCr) as well as localized MM differences in the human brain compared to MC. This may affect comparison of literature values between MRS studies, which use different WS techniques. Importantly, the MM spectrum included in the LCModel basis set must be measured with the same WS scheme as the metabolite spectrum to have reliable quantification. This water‐mediated MT effect on metabolites is not observed with shorter water saturation (e.g., single WS pulse) and likely becomes more pronounced with longer water saturation time.

## Funding

This work was supported by the National Institutes of Health (1S10OD017974‐01, P30 NS076408, P41 EB027061, and R01 EB030000).

## Supporting information


**Figure S1:** Mean and SD semi‐LASER spectra (*T*_R_/*T*_E_ = 3000/28 ms, 64 averages) acquired from all subjects using MC and MC with a single WS pulse (MC + WS1) water suppression schemes. No visible difference in the spectral pattern was observed. For display purposes, a Gaussian weighting of 0.12 s was applied to all spectra.
**Figure S2:** Overlay of mean metabolite and macromolecule semi‐LASER spectra acquired using MC, VAPOR and MC + WS1 WS techniques. The corresponding difference spectra are also shown, scaled by a factor of two. The metabolite difference spectrum between MC and VAPOR shows clear residuals at two tCr peaks. The macromolecule difference spectrum between MC and VAPOR shows non‐uniform residual across the spectral range. No obvious difference was observed in the difference spectrum between MC and MC + WS1.
**Figure S3:** Mean concentration (mM) of metabolites measured from five participants using the three WS schemes; namely VAPOR, MC + WS1 and MC. * indicates significance after FDR correction (adjusted *p* < 0.05) between WS techniques. Metabolite spectra acquired using VAPOR were quantified using the VAPOR MM basis set while the MC datasets were quantified using the MC MM basis set. This was based on the fact that no difference in spectrum was found between MC and MC + WS1.
**Table S1:** Number of averages used for macromolecule (MM) measurements across subjects. Due to scan‐time limitations and subject comfort considerations, the numbers of averages varied between subjects, as both metabolite and MM spectra were acquired using VAPOR and MC water suppression techniques.
**Table S2:** MRSinMRS checklist.

## Data Availability

The data that support the findings of this study are openly available in UMN DRUM at https://hdl.handle.net/11299/278916.
