# Supplementary material for: Effect of Water Suppression and Metabolite Cycling on Quantification of 1H MRS Spectra in the Human Brain at 3 Tesla
Source: Magn Reson Med. 2026 Mar 19;96(2):542–7. doi: 10.1002/mrm.70352 (PMC13269189; doi:10.1002/mrm.70352)
Supplement: Supplementary file 1 — Figure S1: Mean and SD semi‐LASER spectra (T R/T E = 3000/28 ms, 64 averages) acquired from all subjects using MC and MC with a single WS pulse (MC + WS1) water suppression schemes. No visible difference in the spectral pattern was observed. For display purposes, a Gaussian weighting of 0.12 s was applied to all spectra. Figure S2: Overlay of mean metabolite and macromolecule semi‐LASER spectra acquired using MC, VAPOR and MC + WS1 WS techniques. The corresponding difference spectra are also shown, scaled by a factor of two. The metabolite difference spectrum between MC and VAPOR shows clear residuals at two tCr peaks. The macromolecule difference spectrum between MC and VAPOR shows non‐uniform residual across the spectral range. No obvious difference was observed in the difference spectrum between MC and MC + WS1. Figure S3: Mean concentration (mM) of metabolites measured from five participants using the three WS schemes; namely VAPOR, MC + WS1 and MC. * indicates significance after FDR correction (adjusted p < 0.05) between WS techniques. Metabolite spectra acquired using VAPOR were quantified using the VAPOR MM basis set while the MC datasets were quantified using the MC MM basis set. This was based on the fact that no difference in spectrum was found between MC and MC + WS1. Table S1: Number of averages used for macromolecule (MM) measurements across subjects. Due to scan‐time limitations and subject comfort considerations, the numbers of averages varied between subjects, as both metabolite and MM spectra were acquired using VAPOR and MC water suppression techniques. Table S2: MRSinMRS checklist. [file MRM-96-542-s001.docx]

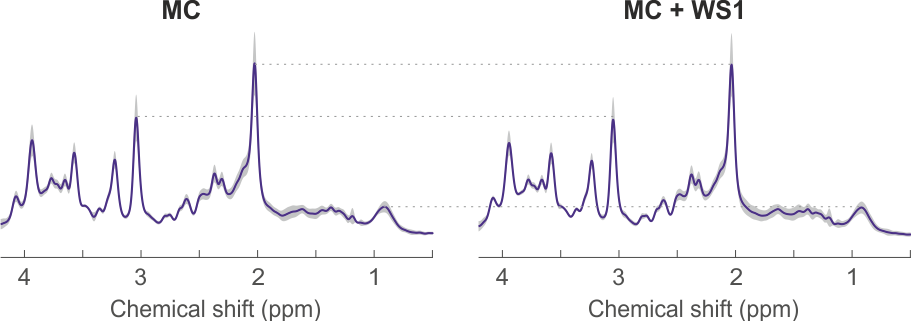


**Figure S1:** Mean and SD semi-LASER spectra (T_R_/T_E_=3000/28 ms, 64 averages) acquired from all subjects using MC and MC with a single WS pulse (MC+WS1) water suppression schemes. No visible difference in the spectral pattern was observed. For display purposes, a Gaussian weighting of 0.12 s was applied to all spectra.


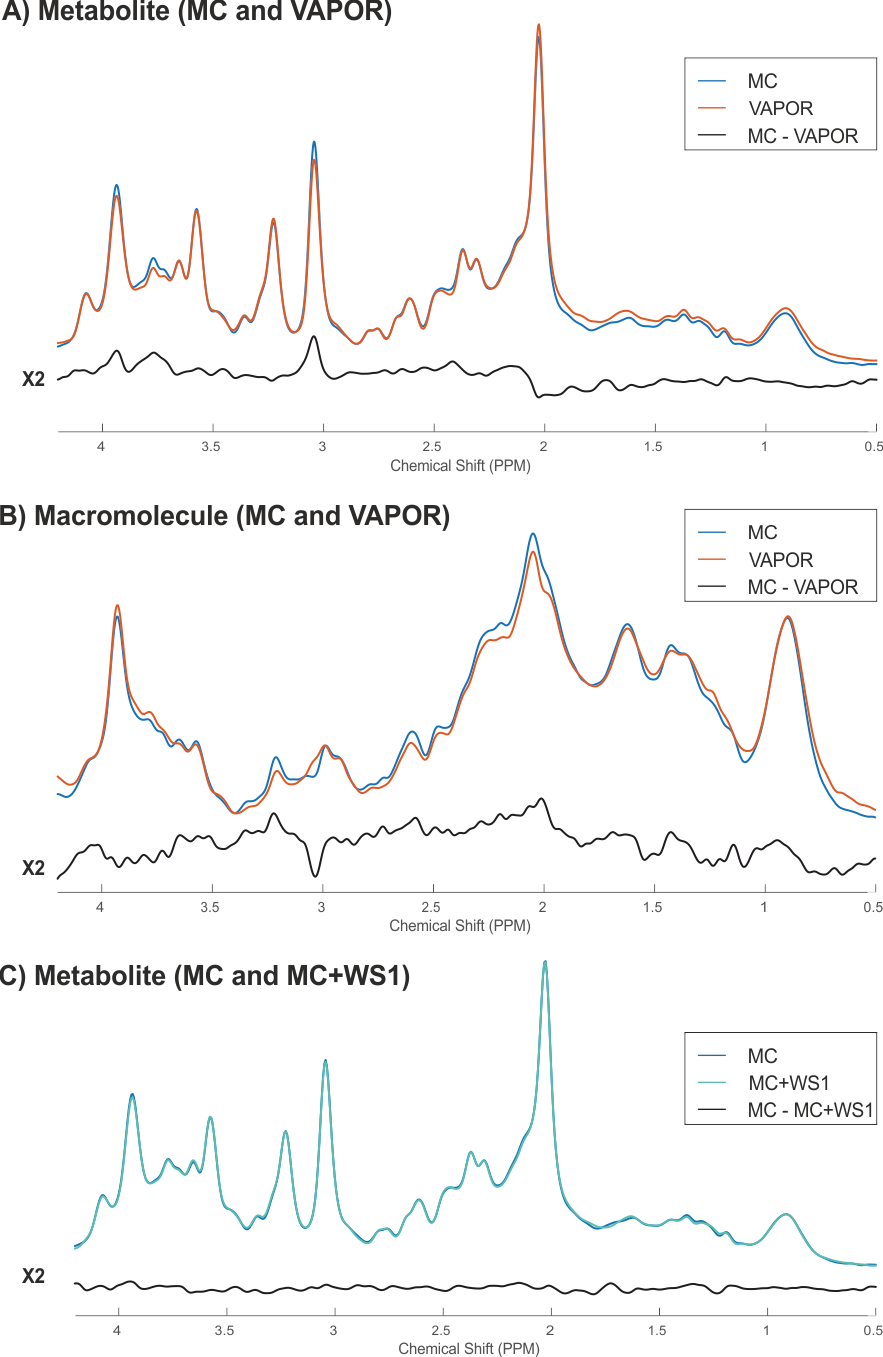


**Figure S2:** Overlay of mean metabolite and macromolecule semi-LASER spectra acquired using MC, VAPOR and MC+WS1 WS techniques. The corresponding difference spectra are also shown, scaled by a factor of two. The metabolite difference spectrum between MC and VAPOR shows clear residuals at two tCr peaks. The macromolecule difference spectrum between MC and VAPOR shows non-uniform residual across the spectral range. No obvious difference was observed in the difference spectrum between MC and MC+WS1.


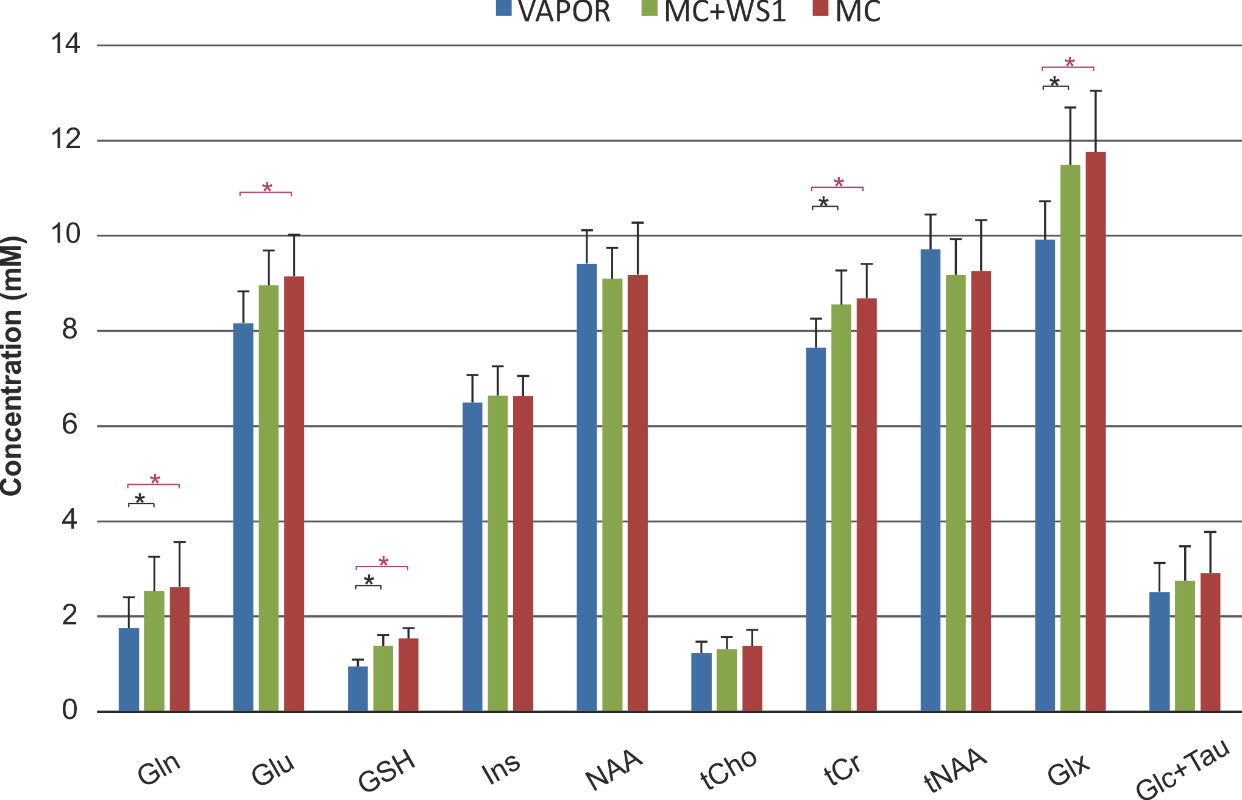


**Figure S3:** Mean concentration (mM) of metabolites measured from five participants using the three WS schemes; namely VAPOR, MC+WS1 and MC. * indicates significance after FDR correction (adjusted *P* < 0.05) between WS techniques. Metabolite spectra acquired using VAPOR were quantified using the VAPOR MM basis set while the MC datasets were quantified using the MC MM basis set. This was based on the fact that no difference in spectrum was found between MC and MC+WS1.

**Table S1**: Number of averages used for macromolecule (MM) measurements across subjects. Due to scan-time limitations and subject comfort considerations, the numbers of averages varied between subjects, as both metabolite and MM spectra were acquired using VAPOR and MC water suppression techniques.

|  | VAPOR | MC |
| --- | --- | --- |
| S1 | 256 | 256 |
| S2 | 256 | 256 |
| S3 | 160 | 160 |
| S4 | 128 | 128 |
| S5 | 200 | 200 |

**Table S2**: MRSinMRS checklist.

| 1. Hardware |  |
| --- | --- |
| a. Field strength [T] | 3 T |
| b. Manufacturer | Siemens |
| c. Model (software version if available) | Prisma^Fit^ (Syngo VE11C) |
| d. RF coils: nuclei (transmit/receive), number of channels, type, body part | Transmit: body coil  Receive: 32-channel head coil |
| e. Additional hardware | N/A |
| 2. Acquisition |  |
| a. Pulse sequence | Semi-LASER |
| b. Volume of Interest (VOI) locations | posterior cingulate cortex |
| c. Nominal VOI size [cm^3^, mm^3^] | 2 x 2 x 2 cm^3^ |
| d. Repetition Time (TR), Echo Time (TE) [ms, s] | Metabolite scans: TR = 3000 ms; TE=28ms where TE1/TE2/TE3 = 8/11/9 ms  Macromolecule scans: TR = 2500 ms; TE=28ms where TE1/TE2/TE3 = 8/11/9 ms; TI=750 ms |
| e. Total number of Excitations or acquisitions per spectrum  In time series for kinetic studies   1. Number of Averaged spectra (NA) per time-point 2. Averaging method (e.g. block-wise or moving average) 3. Total number of spectra (acquired / in time-series) | Metabolite scans: 64 transients per subject  Macromolecule scans: 128 or 256 transients per subject  Stored as individual FIDs |
| f. Additional sequence parameters (spectral width in Hz, number of spectral points, frequency offsets)  If STEAM:, Mixing Time (TM)  If MRSI: 2D or 3D, FOV in all directions, matrix size, acceleration factors, sampling method | Spectral width = 6 kHz with 2048 complex points  OVS parameters:   - 5.12 ms HS1R40 - Slab thickness (all 6 directions) = 120 mm - gap spacing = 7 mm |
| g. Water Suppression Method | VAPOR with suppression bandwidth of 70 Hz |
| h. Shimming Method, reference peak, and thresholds for “acceptance of shim” chosen | FAST(EST)MAP B0 shimming with 4 iterations |
| i. Triggering or motion correction method  (respiratory, peripheral, cardiac triggering, incl. device used and delays) | N/A |
| 3. Data analysis methods and outputs |  |
| a. Analysis software | MRspa v2 May 2024 |
| b. Processing steps deviating from quoted reference or product | Processing steps for VAPOR metabolite data:   1. Eddy current correction 2. Frequency and phase corrections using cross-correlation 3. Frequency correction using amplitude mode on NAA peak 4. Sum FIDs   Processing steps for MC metabolite data:   1. Frequency correction using cross-correlation 2. Phase correction using maximum intensity 3. Frequency correction using water peak 4. Subtract odd and even FIDs 5. Frequency correction using amplitude mode on NAA peak 6. Sum FIDs 7. Eddy current correction   Processing steps for VAPOR macromolecule data:   1. Eddy current correction 2. Average 4 FIDs 3. Frequency correction followed by phase correction using cross-correlation algorithms (default settings) 4. Frequency correction using amplitude mode on tCr-CH_2_ peak at 3.93 ppm 5. Sum FIDs   Processing steps for MC macromolecule data:   1. Phase correction using maximum intensity 2. Frequency correction using water peak 3. Remove pairs of odd and even corrupted water peaks (if applicable) 4. Subtract odd and even FIDs 5. Frequency correction using amplitude mode on tCr-CH_2_ peak at 3.93 ppm 6. Sum FIDs 7. Eddy current correction |
| c. Output measure  (e.g. absolute concentration, institutional units, ratio) | Metabolite concentrations were quantified using LCModel v6.3-0G and scaled to the unsuppressed water peak |
| d. Quantification references and assumptions, fitting model assumptions | LCModel “CONTROL” parameters:  DKNTMN=0.25  FWHMBA=0.0162  NRATIO=0  NSIMUL=0  NUSE1=5  CHUSE1= 'Cr','PCr','NAA','Glu','Ins'  PPMST=4.2  PPMEND=0.5  RFWHM=2.5  SDDEGZ = 0.00  SDDEGP = 0.00  WSMET='Cr'  N1HMET=3  WSPPM=3.0241 |
| 4. Data Quality |  |
| a. Reported variables  (SNR, Linewidth (with reference peaks)) | SNR of NAA and water linewidth reported |
| b. Data exclusion criteria | No subjects excluded |
| c. Quality measures of postprocessing Model fitting (e.g. CRLB, goodness of fit, SD of residual) | Metabolites with mean CRLB ≤ 20% are reported |
| d. Sample Spectrum | Figures 1, 2, S1 and S2 |
